# Environmental Specification of Pluripotent Stem Cell Derived Endothelial Cells Toward Arterial and Venous Subtypes

**DOI:** 10.3389/fbioe.2019.00143

**Published:** 2019-06-14

**Authors:** Seep Arora, Evelyn K. F. Yim, Yi-Chin Toh

**Affiliations:** ^1^Department of Biomedical Engineering, National University of Singapore, Singapore, Singapore; ^2^Singapore Institute for Neurotechnology (SINAPSE), National University of Singapore, Singapore, Singapore; ^3^Department of Chemical Engineering, University of Waterloo, Waterloo, ON, Canada; ^4^Biomedical Institute for Global Health Research and Technology (BIGHEART), National University of Singapore, Singapore, Singapore; ^5^NUS Tissue Engineering Program, National University of Singapore, Singapore, Singapore

**Keywords:** human pluripotent stem cells, endothelial cells, substrate topography, shear stress, arterial specification, functional maturation, environmental cues

## Abstract

Endothelial cells (ECs) are required for a multitude of cardiovascular clinical applications, such as revascularization of ischemic tissues or endothelialization of tissue engineered grafts. Patient derived primary ECs are limited in number, have donor variabilities and their *in vitro* phenotypes and functions can deteriorate over time. This necessitates the exploration of alternative EC sources. Although there has been a recent surge in the use of pluripotent stem cell derived endothelial cells (PSC-ECs) for various cardiovascular clinical applications, current differentiation protocols yield a heterogeneous EC population, where their specification into arterial or venous subtypes is undefined. Since arterial and venous ECs are phenotypically and functionally different, inappropriate matching of exogenous ECs to host sites can potentially affect clinical efficacy, as exemplified by venous graft mismatch when placed into an arterial environment. Therefore, there is a need to design and employ environmental cues that can effectively modulate PSC-ECs into a more homogeneous arterial or venous phenotype for better adaptation to the host environment, which will in turn contribute to better application efficacy. In this review, we will first give an overview of the developmental and functional differences between arterial and venous ECs. This provides the foundation for our subsequent discussion on the different bioengineering strategies that have been investigated to varying extent in providing biochemical and biophysical environmental cues to mature PSC-ECs into arterial or venous subtypes. The ability to efficiently leverage on a combination of biochemical and biophysical environmental cues to modulate intrinsic arterio-venous specification programs in ECs will greatly facilitate future translational applications of PSC-ECs. Since the development and maintenance of arterial and venous ECs *in vivo* occur in disparate physio-chemical microenvironments, it is conceivable that the application of these environmental factors in customized combinations or magnitudes can be used to selectively mature PSC-ECs into an arterial or venous subtype.

## Introduction

Endothelial cells (ECs) can either be derived as primary ECs from donors or differentiated from stem cells for various clinical applications. Primary ECs are limited by donor availability and are subjected to donor-dependent variabilities (Wong et al., 2012). One of the upcoming alternative EC source for clinical applications is human pluripotent stem cell derived endothelial cells (PSC-ECs). The proposed clinical applications of PSC-ECs include endothelialization of tissue engineered vascular grafts and cell therapy for myocardial ischemia or peripheral arterial occlusive disease (PAOD) (Leeper et al., 2010; Patterson et al., 2012; Reed et al., 2013). Multiple studies have reported the successful differentiation of ECs from different PSCs, including embryonic stem cells (ESCs) and induced pluripotent stem cells (iPSCs), by using cytokine cocktails to mimic the vascular developmental program (Levenberg et al., 2002; McCloskey et al., 2005; Leeper et al., 2010; Glaser et al., 2011; Li et al., 2011; Adams et al., 2013; Rufaihah et al., 2013; Tan et al., 2013; Sivarapatna et al., 2015; Zhang et al., 2017). In most studies, the differentiated cells are isolated based on the expression of a ubiquitous endothelial marker such as CD-31 (Rufaihah et al., 2013; Sivarapatna et al., 2015) or VE-Cadherin (Adams et al., 2013), which cannot discern between arterial and venous phenotypes. As a result, PSC-ECs express both arterial and venous markers (Rufaihah et al., 2013; Sriram et al., 2015). The extent of arterial-venous heterogeneity in PSC-ECs remains uncharacterized, which in turn prohibits the PSC-EC population to be sorted by fluorescence activated cell sorting (FACS) (Rufaihah et al., 2013). Evidences from previous reports on venous ECs mismatch when engrafted at arterial sites (Kudo et al., 2007) suggest that the phenotypes and functions of engrafted ECs should match those of the host tissues in order to achieve long term clinical efficacy. This implies that a PSC-EC population that is phenotypically and functionally heterogeneous may not be very useful from a clinical application point-of-view (Kudo et al., 2007; Muto et al., 2011). However, existing research in the derivation of a more homogenous PSC-EC subtype has been limited so far. Therefore, there is a need to devise more effective strategies that can selectively mature PSC-ECs into arterial and venous subtypes for their successfully deployment in various clinical applications.

Since the development and maintenance of arterial and venous ECs *in vivo* occur in disparate physio-chemical microenvironments, with differences in growth factor concentrations, cell adhesion molecules, shear stress magnitudes, oxygen concentrations and basement membrane architectures (dela Paz and D'Amore, 2009; Liliensiek et al., 2009; Sivarapatna et al., 2015), it is conceivable that the application of these environmental factors in customized combinations or magnitudes can be used to selectively mature PSC-ECs into an arterial or venous subtype. This review aims to provide a framework as well as highlight opportunities to advance current PSC-EC differentiation protocols from EC lineage commitment to arterial-venous specification. To this end, we will first discuss the developmental and environmental differences that exist between arterial and venous ECs *in vivo*. This would provide important insights into engineering arterial or venous-enriching microenvironments *in vitro* during the derivation of PSC-ECs. The review will discuss current methods of PSC-ECs derivation and their limitations in generating enriched arterial or venous EC populations. Finally, we will summarize and discuss various biochemical and biophysical strategies, which have been previously employed or are potentially useful for obtaining pure arterial and venous subtypes from PSC-ECs.

## The Potential and Challenges of PSC-ECs in Clinical Applications

Cardiovascular diseases are a common cause of mortality worldwide, accounting for 31% deaths globally (WHO, 2017), out of which, the prevalence of arterial complications is higher as compared to venous pathologies. Nonetheless, the incidence of these venous disorders is increasing, which may lead to a demand for venous ECs to vascularize the damaged venous endothelium (ISTH Steering Committee for World Thrombosis Day, 2014).

Arterial stenosis, which progresses into a variety of clinical cardiac anomalies, require bypass surgeries using vascular grafts. Currently, autologous saphenous vein is being used as the “gold standard” conduit for bypass surgeries (DiMuzio and Tulenko, 2007). Despite being autologous and immunologically compatible, saphenous vein grafts face adaptation problems due to the microenvironmental differences that exist between an artery and a vein (Muto et al., 2010). Most vein grafts remodel within the first month after the surgery; grafts that do not undergo any adaptation have a 13-fold higher chance of failure (Owens et al., 2015). Current research suggests that this might be due to the limited remodeling capacity of terminally differentiated venous ECs in an arterial environment. The adaptation of the venous endothelium to the arterial environment is determined by a switch in the expression of biomolecular modulators that maintain the venous endothelium to those that maintain the arterial endothelium. For instance, Muto et al. (2010, 2011) demonstrated that the expression of Ephrin type B receptor 4 (EphB4) is responsible for the maintenance of the venous phenotype. The venous graft can adapt to an arterial microenvironment when EphB4 expression is lost, whereas a persistent expression of EphB4 prevents the graft from remodeling in the new arterial environment (Muto et al., 2011). Similar previous studies demonstrated that a loss of EphB4 expression in venous EC inside a vein graft under high shear stress conditions may not necessarily be accompanied by a concomitant upregulation of arterial EphrinB2, resulting in an incomplete adaptation (Kudo et al., 2007; Yang et al., 2013).

Tissue engineered vascular grafts (TEVGs) are proposed as engineered alternatives to vein grafts to replace occluded peripheral and coronary vessels (Catto et al., 2014). TEVGs are often constructed from biomaterials and will require *a priori* endothelialization with isolated ECs before implantation into patients. One common source of ECs would be primary ECs directly isolated from patients. However, these ECs are limited by their availability and suffer from batch-to-batch variations. More importantly, the patency of TEVGs even after endothelialization is lower as compared to autologous vein grafts, which can be contributed by multiple factors, including the cell type being used (Pashneh-Tala et al., 2015). TEVGs undergoing clinical trials have mostly utilized autologous venous ECs (Tiwari et al., 2001), which may potentially mal adapt when transplanted into an arterial environment similar to vein grafts.

The above studies relating graft patency to the maladaptation of transplanted ECs at the host environment site suggest that the ability to match or remodel EC phenotypes to their transplanted host microenvironment is important to achieve positive outcomes in clinical applications. They also allude to the fact that the plasticity of mature ECs is limited (Kudo et al., 2007). Therefore, vascular grafts and similar vascular clinical applications need alternative EC sources, which have better endothelial plasticity and will acclimatize to the transplanted environment more easily. Current research into PSC-ECs may offer a promising alternative to the primary ECs. PSCs have higher expansion capacity and increased plasticity, which would support their maturation into an arterial or venous subtype in response to a presented microenvironment (Hatano et al., 2013; Rufaihah et al., 2013; Sivarapatna et al., 2015).

Many studies have reported the successful derivation of ECs from PSCs, which are phenotypically and functionally reminiscent of primary ECs but tend to express both arterial and venous molecular markers. PSC-ECs have been evaluated by multiple groups for cell therapy applications, such as ischemic tissue repair (Sone et al., 2007; Huang et al., 2010; Rufaihah et al., 2013) and myocardial infarction (Li et al., 2009; Prado-Lopez et al., 2010; Kim et al., 2011; Zhang et al., 2017). PSC-ECs can successfully form micro-vessels in animal models, but their integration with the host vasculature is highly inconsistent (Levenberg et al., 2002; Ferreira et al., 2007; Wang et al., 2007). Huang et al. (2010) demonstrated that the introduction of PSC-ECs enhanced perfusion and neovascularization of an ischemic hindlimb in a mouse model; while Prado-Lopez et al. (2010) observed an improvement in cardiac function when ECs derived from ESCs were introduced at a myocardium infarcted site in rat models. Although these studies suggest that PSC-ECs can have therapeutic efficacy, there are emerging reports that the use of a purer PSC-EC subtype leads to better performance over a heterogenous PSC-EC population. For instance, Rufaihah et al. (2013) observed enhanced neovascularization ability by arterial enriched PSC-ECs as compared to a heterogeneous population of PSC-ECs when injected at an ischemic site. Similarly, Zhang et al. (2017) showed an improved cardiovascular function with arterial induced PSC-ECs in comparison to venous induced PSC-ECs, when introduced at the myocardial infarct site. However, *in vivo* applications of PSC-ECs in TEVGs is yet to be demonstrated; although a biomimetic vascular graft has been synthesized using co-culture of iPSC-EC and iPSC-derived smooth muscle cells (SMC) on collagen coated nanofibrils (Nakayama et al., 2015b). The authors demonstrated a two-layered hollow graft with iPSC-ECs longitudinally aligned with an outer iPSC-SMC layer, which showed a reduced inflammatory response *in vitro*. It is likely that the PSC-ECs in such TEVGs would also need to undergo successful remodeling when implanted *in vivo*, as in the case of vein grafts, to retain the graft's patency. Therefore, the understanding and development of bioengineered environments to derive purer arterial or venous PSC-EC subtypes would have greater therapeutic impact.

## Functional and Developmental Differences Between Arterial and Venous Endothelial Cells *in vivo*

**Figure 1:**
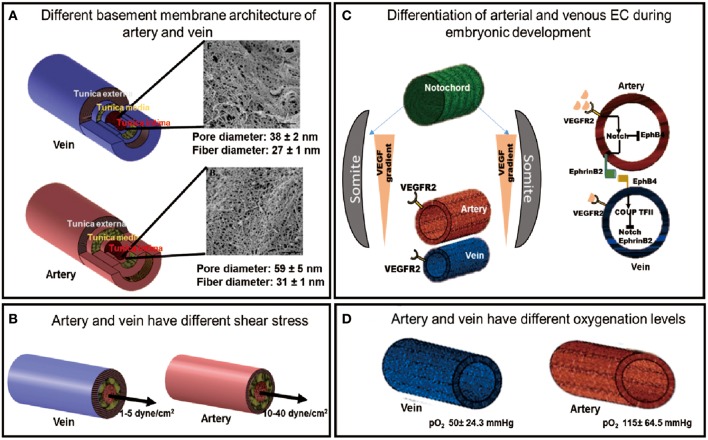
The developmental and physiological functions of arteries and veins are governed by different environmental factors. **(A)** The different basement membrane architectures of the artery and vein [SEM images adapted from Liliensiek et al. (2009), reproduced with permission from Mary Ann Liebert, Inc., New Rochelle, NY]. **(B)** Different shear stress experienced by arteries and veins. **(C)** Different differentiation pathways of artery and vein during embryonic development. **(D)** Artery and vein experience different oxygenation levels of the blood.

*In vivo*, arterial and venous ECs are phenotypically and functionally distinct due to differences in their developmental program and the microenvironment they reside in (Figure 1). An understanding of these differences will provide guiding principles to design strategies to generate and identify an enriched arterial or venous PSC-EC population.

### Functional Differences Between Arterial and Venous ECs

The vascular system is heterogeneous and consists of arteries, veins and capillaries. Each vessel differs from each other in its structure, function and its constituent cell types. Both artery and vein possess three layers: *tunica intima, tunica media*, and *tunica adventitia* with varying thickness and composition of each layer between the two vessel types. *Tunica adventitia*, which provides the structural support to the vessel, is the thickest layer in the vein constituting most of the venous wall whereas, it is much thinner in the arteries. The *tunica media*, which confers elasticity to the vascular wall, is the thickest in arteries with multiple layers of smooth muscle cells, making it more elastic and thus vasoactive than veins. The *tunica media* of vein is not clearly defined, consequently veins have much thinner walls and are more malleable. The innermost layer, *tunica intima*, contains the ECs lining the vessel wall. The endothelium of each vessel resides on the basement membrane of the *tunica intima*, which has been shown to have distinct architectures in artery and vein (Figure 1A) (Liliensiek et al., 2009). These structural differences between arteries and veins regulate their different functions.

Vasomotor tone is the ability of the blood vessel to change its volume in response to varying blood pressure, which is also regulated by the shear stress it experiences. High shear stresses in the artery (10–40 dyne/cm^2^) regulate the secretion of vasodilating and constricting factors, such as Nitric oxide (NO) and endothelin, respectively, to actively modify the vessel volume in response to hemodynamic pressure by acting on the smooth muscle cells in the *tunica media*. In contrast, veins experience passive blood flow with lower flow rates (1–5 dyne/cm^2^) (Figure 1B), which corresponds to the lower vasomotor tone of a diminutive *tunica media* (LaBarbera, 1990; dela Paz and D'Amore, 2009; Prado-Lopez et al., 2010). The two vessel types along with a different shear stress also experience different oxygenation levels which is determined by their different functions (Figure 1D).

The arterial and venous endothelium also varies in their intercellular adhesiveness, which is regulated by tight junctions (*zona occludens*), adherence junction (*zona adhaerens*), and gap junctions consisting of connexin proteins (dela Paz and D'Amore, 2009). Venous endothelium has been reported to be more permeable due to the presence of weak *tight* and *adherence* junctions (Geenen et al., 2015). Conversely, the arterial endothelium possesses much stronger *adherences* and *tight* junctions, and hence are less permeable to soluble molecules. Due to the low flow rate it experiences and higher permeability, venous endothelium is the major site of leucocyte trafficking. The propensity for activated monocytes to migrate across the permeable venous endothelium is indicative of its enhanced inflammatory behavior (dela Paz and D'Amore, 2009; Geenen et al., 2015; Cahill and Redmond, 2016).

### Genetic Determinants of Arterial-Venous EC Specification

The major receptor-ligand association for the genetic determination of ECs involves the Eph family transmembrane ligand, EphrinB2, and its receptor, EphB4. Chicken Ovalbumin upstream promoter-transcription factor II (COUP-TFII) is a key transcription factor, which determines venous specification of ECs, and inhibits the expression of the arterial marker, Notch1 (dela Paz and D'Amore, 2009). During fetal development, arterial-venous specification pathway begins with the spatial patterning of Sonic hedgehog (SHH), which is expressed in the notochord of developing embryos. SHH induces the somites to express vascular endothelial growth factor (VEGF). The activation of VEGF in the dorsal aorta leads to downstream activation of the Notch signaling pathway, leading to arterial ECs development. Reduced SHH signal experienced by the posterior cardinal vein results in the attenuation of VEGF signaling, which results in venous specification. Activation of Notch pathway in the arterial-fated cells leads to the eventual expression of the EphrinB2 ligand, whereas in venous-fated cells, EphB4 receptor is expressed due to the activation of COUP-TFII (Figure 1C) (dela Paz and D'Amore, 2009; Tan et al., 2013). The arterial endothelium is marked by the expression of EphrinB2, Neuropilin-1 (NRP-1), Notch family markers like Notch1-4 and their receptor, Delta like ligand (Dll) and Jagged; whereas the venous endothelium is characterized by the presence of EphB4, NRP-2 and the transcriptional factor COUP-TFII (Wang et al., 1998; Hamada, 2003; Yang et al., 2013).

### Environmental Factors for Maintaining Arterial and Venous Phenotypes *in vivo*

Besides genetic factors, arterial and venous specification during development is also dependent on environmental cues around it (Adams, 2003). Although arterial and venous ECs in a mouse embryo are molecularly distinct even before the onset of hemodynamic flow (Wang et al., 1998), they exhibit plasticity, which is regulated by external stimuli, such as the spatial location of these cells, the hemodynamic flow and oxygen concentration (Delphine Moyon et al., 2001; le Noble et al., 2004). Hemodynamic flow has been shown to strongly influence the induction and maintenance of arterial and venous ECs phenotypes during development. For instance, Delphine Moyon et al. (2001) demonstrated environmental dependence of arterial-venous phenotype by transplanting vessel sections from various stages of quail embryo development (E2 to E11), into arterial/venous host sites in a chick embryo. Before E7 stage of development, the artery and vein markers were co-expressed in both vessel types in the chick host coelom, whereas beyond E7, the artery or vein markers were expressed exclusively in host artery and vein, respectively. This indicated that there exists a developmental window whereby primitive ECs are amenable to environment-induced phenotype switching. Similarly, le Noble et al. (2004) demonstrated that the presence or absence of blood flow can induce the developing vasculature to switch between an arterial and venous phenotype in a chick embryo. The study showed that plasticity of ECs leads to the emergence of venous marker expression in originally arterial location upon the redirection of the blood flow. When flow is completely absent due to vessel ligation, the expression of both arterial and venous markers is abolished. This demonstrated that the endothelial plasticity and maintenance of its phenotype is regulated by blood flow.

Oxygen concentration is another important factor in regulating vascular development. Diffusion is the predominant mode of oxygen transfer in an embryo, which becomes limited during gastrulation leading to a lower oxygen concentration in the embryo. Hence, subsequent vasculogenesis occurs in a hypoxic environment. Low oxygen concentration leads to the upregulation of hypoxia inducing factor (HIF), which in turn regulates the expression of arterial-promoting VEGF and NICD (Notch intracellular domain) (Lanner et al., 2013). Although there is no direct report on the regulation of venous EC specification by oxygen tension, current literature points to the existence of an oxygen gradient in an embryo due to diffusion from the surface. Tissue regions subjected to relatively higher oxygen concentration may result in the suppression of HIF and VEGF regulation pathways, which leads to lower VEGF concentration, and consequently resulting in venous EC specification.

Based on the observations from the vein graft remodeling studies and EC plasticity during embryonic development, it can be inferred that, to some extent, EC phenotypes are highly plastic and responsive to their environment during the nascent stage of their development. This plasticity diminishes as the ECs mature.

## Environmental Factors for Modulating EC Arterial-Venous Specification *in vitro*

A large number of differentiation protocols have been reported to generate ECs from hPSCs to date, although they largely follow the developmental progression of ECs in embryonic vasculatures (summarized in Table 1). Typically, PSCs are induced into a mesodermal lineage either by embryoid body (EB) formation, coculture with other stromal cells, and increasingly, direct induction with small molecules and growth factors as a 2D monolayer before various biochemical factors [e.g., VEGF and bone morphogenetic protein (BMP)] are applied to direct the mesodermal progenitors to differentiate along an endothelial lineage. The efficiency of a direct induction protocol is the highest among the three protocols, with a derivation efficiency ranging from 30 to 99% (Kane Nicole et al., 2010; Lian et al., 2014; Wu et al., 2015; Wang et al., 2016; Rosa et al., 2019). The EB method yields between 2 and 50% PSC-ECs (Levenberg et al., 2002; Ferreira et al., 2007; Goldman et al., 2009; Nourse et al., 2010; Adams et al., 2013; Rufaihah et al., 2013), whereas the feeder layer method yields 1–20% of PSC-ECs (Vodyanik et al., 2005; Choi et al., 2009; Kelly and Hirschi, 2009; Taura et al., 2009), which is the lowest among the three differentiation approaches discussed here. The derived EC population is subsequently sorted by a pan-endothelial marker, e.g., CD-31 (Nourse et al., 2010; White et al., 2013) or VE-Cadherin (Li et al., 2007; Adams et al., 2013) to separate them from non-EC population.

**Table 1:** Derivation and characterization of ECs from PSCs classified by culture method.

**Cell source**	**Induction protocol with supplements**	**Markers expressed**	**Functions tested**	**EC selection method**	**References**
hESC	EB formation None	vWF, CD31, VE-Cadherin	Matrigel tube formation assay *In vivo* angiogenic assay	CD31^+^ cell sorting	Levenberg et al., 2002
hESC	EB formation FBS supplemented VEGF (50 ng/ml)	VE-Cadherin, vWF, CD-31	Acetylated low density lipoprotein (Ac-LDL) uptake *In vivo* angiogenic potential	CD34^+^ cell sorting	Ferreira et al., 2007
hESC	EB formation IL-3, IL-6, G-CSF, Flt3L, SCF, VEGF (10–50 ng/ml), and BMP4 (10 ng/ml)	vWF, CD31, CD144	TNFα stimulated ICAM-1 expression Ac-LDL uptake Matrigel tube formation assay	CD144^+^ and KDR^+^ cell sorting	Goldman et al., 2009
hESC	EB formation VEGF (0–100 ng/ml)	CD31, VE-Cadherin, vWF	Ac-LDL uptake Nitric oxide synthase expression TNF-α stimulated ICAM-1, VCAM-1 expression *In vitro* and *in vivo* angiogenic potential	CD31^+^ cell sorting	Nourse et al., 2010
hiPSC	EB formation BMP4 (50 ng/ml), FGF2 (20 ng/ml), and VEGF (50 ng/ml)	VE-Cadherin, VWF, CD31	Ac-LDL uptake Matrigel tube formation assay	Isolation and media enrichment	Feng et al., 2010
mESC	EB formation VEGF (50 ng/ml)	VE-Cadherin, vWF, and eNOS	Ac-LDL uptake *In vitro* and *in vivo* angiogenic potential	VE-Cadherin^+^ cell sorting	Huang et al., 2010
hiPSC	EB formation FCS	VE-Cadherin, VEGFR2, CD31, eNOS	Ac-LDL uptake. Matrigel tube formation assay TNF α & IL-1β stimulated ICAM-1 expression	VE-Cadherin^+^ cell sorting	Adams et al., 2013
hiPSC	EB formation VEGF (50 ng/ml), BMP4 (50 ng/ml)	eNOS, vWF, VEGFR2	Ac-LDL uptake Matrigel tube formation assay TNFα stimulated ICAM1 expression	CD31^+^ cell sorting	Rufaihah et al., 2013
hiPSC	EB formation in FBS and BMP4 (20 ng/ml) FBS, VEGF (50 ng/ml)	CD31, VE-Cadherin, vWF, eNOS, VEGFR2	Ac-LDL uptake Matrigel tube formation assay	CD31^+^ cell sorting	Sivarapatna et al., 2015
hiPSC	EB formation VEGF (50 ng/ml), BMP4 (50 ng/ml)	CD31, VE-Cadherin, eNOS	Ac-LDL uptake Matrigel tube formation assay	VE-Cadherin^+^ cell sorting	Nakayama et al., 2015b
mESC	Co-culture with OP9 feeder layer FCS	VEGFR2, VE-Cadherin, CD31, CD34	None	VEGFR2^+^ cell sorting	Hirashima et al., 1999
hESC	Co-culture with OP9 feeder cells None	VEGFR2 (showed similarity to bone marrow hematopoietic progenitors)	None	CD34^+^ cell sorting	Vodyanik et al., 2005
hESC	Mouse embryonic fibroblasts with FB None	CD31, VE-Cadherin, vWF, VEGFR2, EphB4, EphrinB2	Ac-LDL uptake Matrigel tube formation assay	CD34^+^ cell sorting	Wang et al., 2007
hESC	Co-culture with OP9 feeder layer FBS	CD31, VE-Cadherin	3D matrix tube formation assay Ac-LDL uptake	CD31^+^, VE-Cadherin^+^ cell sorting	Kelly and Hirschi, 2009
hiPSC	Co-culture with OP9 feeder layer FBS	VE-Cadherin	Matrigel tube formation assay	CD31^+^ cell sorting	Choi et al., 2009
hiPSC	Co-culture with OP9 feeder layer None	CD31, eNOS	Matrigel tube formation assay	VEGFR2^+^, VE- Cadherin^+^ cell sorting	Taura et al., 2009
hESC	Fibroblast ECM substrate Hypoxia (5 and 1% O_2_ concentrations)	CD31, VE-Cadherin, CD34, VEGFR2	3D matrix tube formation assay *In vivo* transplant for myocardial infarction rat model	None	Prado-Lopez et al., 2010
hESC	Direct Differentiation with FGF2, fibroblast conditioned media Hydrocortisone, human EGF, human FGF, heparin	VEGFR2, CD31, VE-Cadherin	Nitric Oxide (NO) production, *In vitro* wound closure capacity *In vitro* and *in vivo* tube formation capability	-	Kane Nicole et al., 2010
hESC/hiPSC	Direct Differentiation CHIR99021 (6–10 μM), 60 μg/ml ascorbic acid VEGF	CD31, VE-Cadherin, vWF	Ac-LDL uptake Matrigel tube formation assay TNFα mediated immune response	CD34^+^ magnetic sorting	Lian et al., 2014
hESC/hiPSC	Mesodermal induction Y-27632 (10 mM) VTN (250 ng/cm^2^) CHIR99021 (3 μM) ActivinA (2 ng/ml) PVA coating VEGF-A (10–20 ng/ml)	CD31, CDH5	*In vitro* and *in vivo* tube formation capability	CD31^+^ cell sorting	Wu et al., 2015
hiPSC	Direct Differentiation VEGF (upto 80 ng/ml)	UEA-1, vWF, CD31, VE-Cadherin, VEGFR2	Ac-LDL uptake TNF α stimulated ICAM-1 expression ZO-1 expression Matrigel tube formation assay Cell attachment under flow	CD31^+^ cell sorting	Belair et al., 2015
hiPSC	Direct Differentiation Stage I FBS, BMP4 (50 ng/ml), ActivinA (10 ng/ml), VEGF (50 ng/ml), FGF2 (50 ng/ml)	vWF, CD31	Matrigel tube formation assay TNF α stimulated monocyte adhesion Cell attachment under flow	CD31^+^ cell sorting	Wang et al., 2016
	Stage II VEGF (50 ng/ml), FGF2 (50 ng/ml), Y27632 (10 μM), SB431542 (10 μM)				
hESC/hiPSC	Direct Differentiation GSK3β inhibition CHIR990221 (3 μM) VEGFA, EGF, DLL4, Heparin, FGF2 (Notch Activation)	vWF, VE-Cadherin	NO production Matrigel tube formation assay Ac-LDL uptake *In vivo* vasculogenesis	VE-Cadherin^+^, CDH5^+^ cell sorting	Lee et al., 2017
hiPSC	Direct Differentiation FGF2 (4 ng/ml)Insulin, ActivinA (125 ng/ml), BMP4 (10 ng/ml), FGF2 (10 ng/ml), VEGF (100 ng/ml), cAMP (1 mM)	CD31, VE-Cadherin, eNOS	Ac-LDL uptake Matrigel tube formation assay	VE-Cadherin^+^ cell sorting	Ikuno et al., 2017
hiPSC	Direct Differentiation WNT activation BMP4 (25 ng/ml), CHIR990221 (7.5 μM) VEGFA (260 ng/ml), Forskolin (2 μM)	VE-Cadherin, eNOS, vWF, NRP1, NRP2, Notch1, EphB4, DLL4	Matrigel tube formation assay Ac-LDL uptake Virus dependent immune response	CD31^+^ magnetic sorting	Olmer et al., 2018
mESC	No mesoderm induction Vascular differentiation VEGF (30 ng/ml), FGF2 (12.5 ng/ml), CHIR99021 (3 μM), BMP4 (12 ng/ml)	VE-cadherin	Matrigel tube formation assay Ac-LDL uptake	VEGFR2^+^ magnetic sorting	Dorsey et al., 2018
hiPSC	Mesoderm induction BMP4 (10/50 ng/ml), FGF2 (20 ng/ml) Endothelial induction VEGF (50 ng/ml), thymosinβ	VEGFR2, CD31, VE-Cadherin, vWF	Ac-LDL uptake Matrigel tube formation assay	CD31^+^ magnetic sorting	Rosa et al., 2019

*hESC, human embryonic stem cells; hiPSC, human induced pluripotent stem cells; mESC, mouse embryonic stem cells; FBS, Fetal bovine serum; FCS, Fetal calf serum; hSCF, human stem cell factor; VTN, Vitronectin*.

Consequently, PSC-ECs generated from current differentiation protocols share the following characteristics: they are (1) reminiscent of immature ECs present in embryonic vasculatures, and (2) phenotypically and functionally heterogenous (Rufaihah et al., 2013; Sivarapatna et al., 2015) because pan-endothelial selection markers do not discern between arterial and venous subtypes. This informs us that efforts to improve the functionality of PSC-ECs for vascular medicine applications must not only mature them but do so in a subtype-specific manner. The nascent state of PSC-ECs also imply that they would have a greater capacity to respond and adapt to their microenvironment than fully matured primary ECs. Thus, *in vitro* culture microenvironments can be fine-tuned to enrich an arterial or venous phenotype based on the intended translational application. Indeed, there have been emerging studies that have attempted to coax these nascent PSC-ECs into arterial or venous subtypes by further maturing them in environments that closely mimic that of an artery or a vein (Jalil et al., 2011). This section will summarize various biochemical and biophysical environmental cues that been shown to enrich PSC-EC population and other stem cell sources into specific subtypes (summarized in Table 2). We will also highlight opportunities for exploiting environmental factors, such as substrate topography and oxygen tension, which are known to modulate mature ECs but are less explored for the maturation of PSC-ECs into specific subtypes.

**Table 2:** Effects of different biochemical and biophysical environmental factors on deriving arterial/venous subtypes from stem cell derived ECs.

**Type of stem cell**	**External factors**	**Arterial characteristics**	**Venous characteristics**	**References**
**BIOCHEMICAL FACTORS**				
hMAPCshAC133^+^	Arterial differentiation: 100 ng/mL VEGF 50 ng/mL VEGF	Upregulation of arterial marker expression EphrinB2, Dll4, and Hey2 Downregulation of arterial marker expression EphrinB2, Dll4, and Hey2	Upregulation of venous marker expression EphB4 No change in venous marker expression	Aranguren et al., 2007
hMSCs	Venous differentiation: 50 ng/ml VEGF Arterial differentiation: 100 ng/ml VEGF	Upregulation of arterial marker expression EphrinB2, Dll4, and Notch4	Upregulation of venous marker expression COUP-TFII and EphB4	Zhang et al., 2008
hiPSC-EC	Venous differentiation: 10 ng/ml VEGF-A Arterial differentiation: 50 ng/ml VEGF-A 0.5 mmol/L 8Br- cAMP	Upregulation of EphrinB2, Notch1, DLL4, Jagged protein expression *In vivo* capillary formation using matrigel plug	Upregulation of EphB4 and Coup TFII protein expression	Rufaihah et al., 2013
hESC-EC	Venous differentiation: 10 ng/ml EGF 20 ng/ml FGF2 Arterial differentiation: 10 ng/ml EGF 20 ng/ml FGF2 10 ng/ml VEGF	NRP1, CXCR4, DLL4 expression *In vivo* angiogenic potential	NRP2 and EphB4 expression *In vivo* angiogenic potential	Sriram et al., 2015
hESC-EC/ hiPSC-EC	Venous differentiation: 50 ng/mL VEGFA, 50 ng/mL BMP4 Insulin Arterial differentiation: 50 ng/ml VEGFA 100 ng/ml FGF2 10 μM SB431542 (TGFβ inhibitor) 5 μM Resveratrol (Notch activator) 5 μM L690 (Inositol monophosphate inhibitor)	Upregulation of EphrinB2, CXCR4, DLL4, HEY, Jagged, Notch1, and Notch4 gene expression NICD protein expression Ac-LDL uptake *In vitro* and *in vivo* angiogenesis Alignment response to shear stress Improved cardiac function in myocardial infarction mouse model	Upregulation of EphB4 and Coup TFII gene expression Higher leucocyte adhesion	Zhang et al., 2017
hiPSC-ECs	Venous differentiation: 10 ng/ml VEGF-A Arterial differentiation: 50 ng/ml VEGF-A	Upregulation of arterial marker expression EphrinB2, Jagged1, Hey2 and Notch4 Higher NO production Low monocyte adhesion ability Higher elongation under shear stress	Upregulation of venous marker expression COUP-TFII and EphB4 Lower NO production High monocyte adhesion ability	Rosa et al., 2019
**SHEAR STRESS**				
EPCs	Rotating disk type flow loading device 0.1–2.5 dyne/cm^2^ for 6 and 24 h	Upregulation of Notch1, Notch3, Hey1, and EphrinB2 mRNA expression	Downregulation of EphB4 and NRP2 mRNA expression	Obi et al., 2009
mESC-EC	Parallel plate flow reactor 1.5–20 dyne/cm^2^	EphrinB2, Notch ligand, and receptors expression increases with increasing shear stress	EphB4 expression reduces with increasing shear stress	Masumura et al., 2009
hiPSC-EC	Bioreactor design Arterial shear: 10 dyne/cm^2^ Venous shear: 5 dyne/cm^2^	Upregulation of arterial marker expression at both shear stresses	Upregulation of venous marker expression at both shear stresses	Sivarapatna et al., 2015
hESC-EC	Multiplex microfluidic device 0.4–15 dyne/cm^2^	Upregulation of Notch1 and EphrinB2 expression beyond ~4 dyne/cm^2^	No significant change in the expression of COUP-TFII and EphB4	Arora et al., 2018
**OXYGEN CONCENTRATION**				
mESC-EC	1.5–2% levels	Upregulation of Notch4, EphrinB2, Dll4, and Hey1 expression at low O2 levels	No significant change in the expression of COUP-TFII and EphB4	Lanner et al., 2013
mESC-EC	Venous differentiation 21% O_2_ levels Arterial differentiation 1% O_2_ levels	Upregulation of Dll4, Notch1, and EphrinB2	Upregulation of COUP-TFII	Tsang et al., 2017
**SUBSTRATE STIFFNESS**				
mEPCs	Venous differentiation: 7 kPa stiffness PDMS 10 ng/ml VEGF 3 ng/ml FGF2 3 ng/ml IGF Arterial differentiation: 128 kPa stiffness PDMS 10 ng/ml VEGF 3 ng/ml FGF2 3 ng/ml IGF	Upregulation of Notch1 and EphrinB2	Upregulation of EphB4	Xue et al., 2017
**CELL-CELL AND CELL-MATRIX INTERACTIONS**				
mESC-EC	Venous differentiation: Immobilized EphrinB2-Fc hydrogel 30 ng/ml VEGF 12.5 ng/ml FGF2 3 μM CHIR99021 Arterial differentiation: Immobilized EphB4-Fc hydrogel 30 ng/ml VEGF 12.5 ng/ml FGF2 12 ng/ml BMP4	Upregulation of Nrp1, Jag1, Dll4, Notch4, and EphrinB2	Upregulation of COUP-TFII and EphB4	Dorsey et al., 2018

*hESC, human embryonic stem cells; hiPSC, human induced pluripotent stem cells; mESC, mouse embryonic stem cells; 8Br-cAMP, 8-bromoadenosine 3':5'-cyclic monophosphate sodium salt; hMAPCs, human multipotent adult progenitor cells; hMSCs, human mesenchymal stem cells; BMP4, Bone morphogenetic protein; EPCs, Endothelial progenitor cells; mEPCs, mouse endothelial progenitor cells*.

### Soluble Factors

Since EC arterial-venous specification is a highly conserved embryonic developmental program that is regulated by the Notch signaling pathway (Iso et al., 2003; Gridley, 2010; Thomas et al., 2013). Notch signaling agonists and antagonists are potent soluble factors that can be easily applied to PSC-EC cultures to directly or indirectly modulate this signaling pathway. VEGF is a potent morphogen in the development of arteries (dela Paz and D'Amore, 2009). VEGF functions upstream of Notch by regulating the expression of Notch ligand, Dll4 (Thomas et al., 2013; Yang et al., 2013). The concentrations of VEGF are crucial for inducting arterial or venous phenotype. The arterial phenotype has been established with a VEGF concentration of 50–100 ng/ml, whereas 10–50 ng/ml resulted in a venous phenotype. For instance, Rufaihah et al. (2013) and Sriram et al. (2015) have shown that 50 ng/ml of VEGF in combination with other factors like cyclic Adenosine monophosphate (cAMP) (Rufaihah et al., 2013), endothelial growth factor (EGF) and basic fibroblast growth factor (bFGF) (Sriram et al., 2015) can preferentially specify PSC-ECs into an arterial subtype. A lower VEGF concentration (10 ng/ml) mimics the physiological situation due to the spatial location of cardinal vein from the notochord, and thus supports venous specification of PSC-ECs (Rufaihah et al., 2013; Sivarapatna et al., 2015). Other growth factors that have been incorporated for arterial or venous induction seem to have a more supplementary role in Notch activation or inhibition since none of the studies have been carried out without VEGF induction. cAMP and adrenomodulin are positive modulators of the Notch signaling pathway, and thus promote an arterial EC phenotype (Atkins et al., 2011). cAMP upregulates the Notch expression indirectly via suppression of COUP-TFII expression (Yurugi-Kobayashi et al., 2006) and also by activation of Phosphoinositide 3 (PI3) kinase which in turn upregulated Notch expression (Yamamizu et al., 2010). On the other hand, inhibition of Notch signaling by exogenous Notch inhibitors, such as α secretase inhibitor attenuates EphrinB2 activation, resulting in a reciprocal increase in EphB4 expression, leading to a venous fate (Lanner et al., 2007; Zhang et al., 2008).

### Shear Stress

Fluid-induced shear stress is an important biophysical factor for regulating EC homeostasis. Disturbances in healthy flow promote vascular complications, including atherosclerosis, tissue ischemia and myocardial infarction (Cahill and Redmond, 2016). An extensive body of literature demonstrates that shear stress aligns ECs morphologically as well as regulates many vasoactive functions and the expression of multiple endothelium-specific genes via mechanotransduction signaling pathways (Feugier et al., 2005; Inoguchi et al., 2007; Masumura et al., 2009; Hattori et al., 2014; Wragg et al., 2014; Sivarapatna et al., 2015). Consequently, shear stress has been depicted to play a vital role in fine-tuning the lineage specification of PSC-ECs. Masumura et al. (2009) investigated the effect of shear stress (5–20 dyne/cm^2^) on the expression of arterial and venous markers in human ESC-derived ECs. They reported a significant upregulation of the arterial marker EphrinB2 and downregulation of the venous marker EphB4 as compared to static control in the presence of 10 dyne/cm^2^ shear stress. The authors postulated that shear stress activates Notch signaling to increase EphrinB2 expression, although the mechano-sensing mechanism is still unknown. Another study by Sivarapatna et al. (2015) investigated two discrete shear stress magnitudes (i.e., 10 dyne/cm^2^ and 5 dyne/cm^2^) for arterial and venous enrichment, respectively. The iPSC-ECs showed an upregulation of arterial markers, EphrinB2 and Notch1, and the venous marker, EphB4, when subjected to both 5 and 10 dyne/cm^2^ of shear stress. However, the venous transcription factor, COUP-TFII, did not demonstrate any significant change in the presence of both shear stress magnitudes. Taken together, it appears that shear stress is positively correlated to an arterial phenotype; although it is still unclear how shear stress will affect venous specification or whether there exists a threshold shear stress magnitude that can toggle between arterial and venous maturation.

Most of the experimental systems to study effects of shear stress on PSC-ECs involve the use of bioreactors or viscometers like parallel plate flow and conical flow reactors. These macroscale setups are limited in their throughput and multiplexing capability because they require large number of cells and reagents and are challenging to operate. This hampers parallel screening of multiple shear stress magnitudes to identify optimal shear stress that will selectively enrich an arterial or venous subtype. Moreover, investigation over a wide range of physiological shear stress magnitudes will shed insights into whether there exists a threshold level where PSC-ECs will switch from a venous phenotype into an arterial one. The adoption of microfluidic technologies to PSC-ECs research will help to circumvent current technical limitations and address knowledge gaps on the effect of shear stress on PSC-ECs. The laminar flow profile in microfluidic system allows a precise control of fluid shear stress experienced by cells via the geometry of the microfluidic channels and the perfusion flow rate (Hattori et al., 2014), leading to a more uniform application of shear stress on the PSC-EC population as compared to macroscale bioreactors. In addition, microfluidic systems can be easily multiplexed to interrogate the effects of multiple shear stress magnitudes simultaneously in a single device (Toh and Voldman, 2011). Our recent study employed a multiplex microfluidic device to simultaneously apply 6 different shear stress magnitudes (0.4–15 dyne/cm^2^) on human ESC derived ECs to investigate their dose-time response to shear stress. The study discovered a threshold shear stress magnitude of ~4 dyne/cm^2^ where an enhancement of Notch1 and EphrinB2 arterial markers could be observed (Arora et al., 2018).

### Oxygen Concentration

Oxygen concentration is another factor that differentiates the arterial and venous endothelium microenvironment. Although the oxygen tension in a healthy mature artery is much higher than a mature vein, vasculogenesis and arterial EC specification appear to be supported by a low oxygen environment. Vascularization in both mature ischemic and early embryonic tissues has been shown to be promoted by oxygen deficiency. Indeed, multiple studies have shown an enhancement in EC differentiation from human or mouse PSC as well as mesenchymal stem cells under hypoxic conditions (Prado-Lopez et al., 2010; Kim et al., 2011; Tsang et al., 2017). Tsang et al. (2017) further investigated the effect of hypoxia on mouse ESC-EC differentiation into an arterial subtype. They showed enhanced expressions of VEGF and Notch1 accompanied by a downregulation of COUP-TFII expression in presence of hypoxia (1% O_2_), which further supports arterial ECs differentiation. Lanner et al. (2013) on the other hand, showed that under hypoxia, endogenous VEGF is not a crucial factor for activating Notch pathway. They tested the effect of hypoxia on Adrenomedullin, which is upregulated independently of the Notch pathway, and acts through the Notch receptor, Dll4 to activate Notch signaling via a positive feedback mechanism. Thus, hypoxia can induce arterial ECs differentiation via a VEGF-independent pathway. From these studies, it is evident that hypoxia as an environmental stimulus can act through Notch signaling pathways to promote arterial specification of PSC-ECs. The timing and duration under which cells are subjected to hypoxic conditions can modulate their arterial differentiation too. Kusuma et al. (2014) tried multiple combinations of hypoxia and normoxia exposure to PSCs and showed that hypoxia (5% O_2_) treatment followed by normoxia increased the pan-EC as well as arterial EC marker expressions in comparison to conditions with consistent normoxia (21% O_2_). This shows that hypoxia is an important regulator of EC differentiation in early embryonic phase.

### Cell-Cell and Cell-Matrix Adhesions

Bidirectional cell-cell interactions between EphrinB2 ligand and EphB4 receptor on arterial and venous cells, respectively, is important for defining the arterio-venous boundary. This leads to a differential expression of EphrinB2 and EphB4 in arterial and venous ECs. The strong specific interaction between this receptor-ligand pair is important for arterial-venous communication since the absence of it results in defective boundaries and vessel malformation (Swift and Weinstein, 2009). Engineered versions of such receptor-ligand interactions have thus been exploited for arterio-venous specification in PSC-ECs. For example, Dorsey et al demonstrated the upregulation of arterial markers in mouse ESCs when cultured on EphB4-Fc immobilized hydrogel. An opposite venous-enhancing effect was observed when the mouse ESCs were cultured on EphrinB2-Fc immobilized hydrogel. The differential expressions of arterial and venous markers were absent when soluble EphrinB2 and EphB4 were introduced to the cells instead, highlighting that the stable EphrinB2-EphB4 ligand-receptor interaction can only be mediated through a cell-adhesive milieu (Dorsey et al., 2018).

The ECM compositions of artery and vein are different. For example, artery is known to have more elastin and collagen than veins because of its need to dilate in response to hemodynamic pressures (Xu and Shi, 2014). Hence, cell-ECM interactions may also play a role in arterio-venous specification of PSC-ECs. Different ECM components have been shown to modulate arterio-venous phenotypes in mature ECs. For instance, Robinet et al demonstrated the effect of elastin peptides on *in vitro* and *in vivo* angiogenesis in chick embryo and micro-vascular ECs (Robinet et al., 2005). In another study, it was shown that collagen promoted vWF expression in both arterial and venous ECs 72 h post shear stress exposure (Geenen et al., 2015). Estrach, Cailleteau et al demonstrated the role of laminin in activation of Notch ligand Dll4 in HUVEC, which was absent when the cells were cultured on collagen and fibronectin (Estrach et al., 2011). However, the effect of different ECM components has not yet been investigated in PSC-ECs. Unlike the EphrinB2-EphB4 cell-cell adhesion, there are no specific ECM proteins that can direct arterio-venous vascular development. It is likely that different combinations of ECM proteins in varying proportions may be required to preferentially direct an arterial or venous fate. High throughput matrix arrays that can spot different ECM proteins in combinatorial fashion and screen for cell responses (Beachley et al., 2015) can be adapted to determine an optimal composite matrix for maturing PSC-ECs into specific subtype. In conclusion, these cell-cell and cell-ECM adhesions are pivotal for regulating mature EC phenotypes and specification of arterial-venous phenotypes. Current studies relating their effects on PSC-EC specification are scant but is a good avenue for future exploration.

### Substrate Topography and Stiffness

*In vivo*, ECs reside on basement membrane which not only differ in their composition, but also their architecture (Liliensiek et al., 2009). Thus, substrate micro/nano-topographical cues have shown to significantly alter a plethora of phenotypic and functional behaviors of mature ECs (Gasiorowski et al., 2010; Morgan et al., 2012; Cutiongco et al., 2018; Kukumberg et al., 2018). These ranged from cytoskeleton rearrangement, cell adherence junctions to global gene expressions modulating EC functions (Table 3). Despite the extensive investigations into how substrate topography modulates mature EC functions (Gasiorowski et al., 2010; Greiner Alexandra et al., 2012), investigation into how topography affects PSC-ECs functions remains relatively less explored. A pilot study by Hatano et al. (2013) demonstrated improved attachment and alignment of mouse ESC-ECs on substrates with nano-wrinkles as compared to flat tissue culture polystyrene substrates. Separate studies by Tan et al. (2018), Nakayama et al. (2015a) and Kim et al. (2017) demonstrated enhanced *in vivo* angiogenesis and arteriogenesis by iPSC-ECs when seeded on scaffolds with more aligned fibers in comparison to the scaffolds with randomly oriented fibers (Table 3). However, the impact of substrate topography on the maturation of PSC-ECs into arterial or venous subtypes is not yet known.

**Table 3:** Effects of substrate topography on mature ECs and PSC-ECs phenotype and functions.

**EC type**	**Topography characteristics**	**Effect**	**References**
HCAEC, dHCAEC	Multi-architectural (MARC) chip with 16 different patterns including gratings, micro-lenses, pillars and holes	Enhanced angiogenic capability of HCAEC and reduced angiogenic capability of dHCAEC Reduced ox-LDL uptake of dHCAEC on topography Reduced immunogenicity of HCAEC on topography Enhanced NOS3 expression in dHCAEC Increased wound healing of dHCAEC on gratings	Cutiongco et al., 2018
HUVEC	Multi-architectural (MARC) chip with 41 different patterns including gratings, micro-lenses, pillars, cones and bumps	Effect on cellular proliferation and cell morphology on different patterns Reduced inflammatory response on micro-lenses	Kukumberg et al., 2018
EA. hy926 (HUVEC cell line)	Pattern: Ridges and grooves Dimension: ridge width-550 nm, depth-600 nm, groove width:550 nm, 1.1 μm, 2.75 μm	Enhanced cellular alignment based on groove ridge axis Downregulation of inflammatory cytokines on patterned substrate	Jeon et al., 2015
HUVEC	Pattern: Ridges and grooves Dimension: pitch- 400, 800, 1,200, 1,600, 4,000 nm Depth: 300 nm	Enhanced cellular alignment and adhesion Enhanced phosphorylation of focal adhesion kinase	Dreier et al., 2013
HUVEC	Pattern: Micropillar Dimension: 1–5.6 μm diameter, 1, 3, 6, 8 μm height, 0.6–15 μm spacing	Enhanced cell elongation and alignment	Dickinson et al., 2012
HUVEC, HAEC	Porous randomly organized substrate mimicking the basement membrane architecture	Reduced inflammatory action in presence of TNFα Increased migration rate of both the cells	McKee et al., 2012
HAEC	Pattern: Ridge and Groove with varying pitches and Holes	Enhanced cellular and nuclear alignment Topography dependent EC migration	Morgan et al., 2012
HUVEC	Pitch: 400 nm Groove to ridge ratio: 1:1 Depth: 300 nm	Upregulation of protein modification genes and downregulates the cell cycle genes Tissue homeostasis	Gasiorowski et al., 2010
mESC	Nano wrinkles and acetone etched surfaces	Enhanced cellular alignment along the topography axis	Hatano et al., 2013
iPSC-EC	Polycaprolactone-gelatin electro spun nanofiber scaffold	Increased survival *in vivo* Increased angiogenesis (arteriole density) *in vivo* Increased VEGF expression of iPSC-ECs *in vivo*	Tan et al., 2018
HMVEC/iPSC-EC	Aligned nanofibrillar collagen scaffold	Cellular elongation on aligned scaffold Higher Integrin α1 expression in ECs on aligned scaffolds Enhanced angiogenesis potential *in vivo* Increased arteriogenesis with iPSC-EC seeded aligned nanofibrillar scaffold	Nakayama et al., 2015a
iPSC-EC	Polycaprolactone and Polyethylene oxide scaffolds: randomly oriented and aligned	Higher vascular network like formation capability on aligned scaffold	Kim et al., 2017

*HCAEC, human coronary artery endothelial cell; dHCAEC, diabetic human coronary artery endothelial cell; ox-LDL, oxidized low-density lipoprotein; NOS3, Nitric oxide synthase 3; HUVEC, human umbilical vein endothelial cell; TNFα, tumor necrosis factor α; HAEC, human aortic endothelial cell; HMVEC, human dermal microvascular endothelial cells*.

It is highly likely that substrate topography would influence the maturation of PSC-ECs into the two subtypes. Topography screening platforms encompassing different topographical architectures, such as the Multi Architecture (MARC) Chip platform (Moe et al., 2012; Cutiongco et al., 2018; Kukumberg et al., 2018) or an algorithm based topographical screening platform (Unadkat et al., 2011) would be a useful tool for identifying topographies that can selectively coax PSC-ECs to mature into an arterial or venous subtype. Once such topographies have been identified and biologically validated for arterial or venous endothelial functional enhancement, it is foreseeable that the topography can be incorporated into both 2D and 3D cell culture scaffolds using 3D printing technologies or lithography-based techniques. These scaffolds can be synthesized using a range of materials, including Poly methyl methacrylate (PMMA), Silicon, Polyurethane, PDMS and even hydrogels, depending on the intended applications (Greiner Alexandra et al., 2012).

Besides the basement membrane, the wall composition of artery and vein as determined by the amount of smooth muscle cells present in the *tunica intima* is also different. Therefore, ECs in arteries and veins also experience different tissue stiffness. An artery consists of more smooth muscle cells due to the need of it to dilate in response to blood pressure leading to higher stiffness of the arterial wall. Culture substrates with tunable stiffness thus, can be used to evaluate whether substrate stiffness can affect arterial-venous specification. For example, Xue et al. (2017) demonstrated the specification of arterial and venous subtypes from endothelial progenitor cells (EPCs) using polydimethylsiloxane (PDMS) substrate of varying stiffness. The authors observed a higher expression of arterial marker EphrinB2 on substrates of 128 kPa, whereas the expression of venous marker EphB4 was higher on softer substrates of 7 kPa. The substrate sensing occurs via Ras/Mek pathway, which consequently regulates the Notch activation similar to effect observed with other environmental cues discussed.

## Conclusion and Future Scope

**Figure 2:**
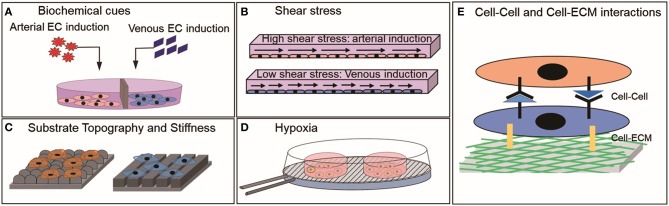
Environmental cues that have shown to influence the physiology of mature ECs or PSC-ECs *in vitro* **(A)** Biochemical factors, **(B)** Shear stress, **(C)** Substrate topography and stiffness, **(D)** Hypoxia, and **(E)** Cell-Cell and Cell ECM interactions.

Arterial and venous ECs are required for multiple clinical and research applications. Deriving them using PSC-ECs will provide a cell source that is not limited by cell number and accessibility. An important hurdle to overcome to realize the practical translation of these cells is to mature them into specific subtypes with functional performance comparable to mature ECs. The strategies discussed here for maturing PSC-ECs into arterial or venous subtypes are predominantly based on a body of work on how environmental factors regulate vascular development. These include both biochemical and biophysical cues (Figure 2), of which the molecular mechanisms underpinning biochemical factor-activated effects are better understood than those of biophysical factors. Most biophysical cues discussed here seem to indirectly regulate the Notch signaling pathway, which in turn promotes an arterial phenotype in PSC-ECs. The exact mechanism for most cases is yet to be explored as to how the cells would sense external cues and translate it into Notch activation or suppression. The clear demarcation between arterial and venous phenotypes was not observed in case of many studies involving biophysical cues, which thus necessitates combining multiple biophysical and biochemical cues to support a more potent specification of the phenotypes. It is highly unlikely that a single factor will be able to drive the PSC-ECs to attain a functional level comparable to primary arterial or venous ECs. Therefore, we foresee that future strategies will employ a combination of biochemical and biophysical factors to activate arterial or venous specification programs like Notch or VEGF-signaling in a concerted manner to derive EC subtypes more efficiently.

## Author Contributions

SA, EY, and Y-CT contributed to the idea conception and the study design. SA did the literature review. SA, Y-CT, and EY wrote the manuscript.

### Conflict of Interest Statement

The authors declare that the research was conducted in the absence of any commercial or financial relationships that could be construed as a potential conflict of interest.
